# Docosahexaenoic acid is comparable to vildagliptin in improving hyperglycemia and pancreatic insulin signaling of diabetic rats via SIRT1/Akt/PI3K pathway

**DOI:** 10.1038/s41598-026-44514-4

**Published:** 2026-04-17

**Authors:** Mariam A. Abo-Saif, Rehab H. Werida, Salma Ashraf Mohamed, Naglaa F. Khedr

**Affiliations:** 1 Department of Biochemistry, Faculty of Pharmacy, Medical Compound, Tanta University, Al-Baher Street, Tanta, P.O 31527 Egypt; 2 Department of Clinical Pharmacy& Pharmacy Practice Department, Faculty of Pharmacy, Damanhour University, Damanhour, 22514 Egypt; 3 Department of Clinical Pharmacy& Pharmacy Practice Department, Faculty of Pharmacy, Alsalam University, AL Gharbia, Kafr AL Zayat, 31611 Egypt

**Keywords:** Docosahexaenoic acid, Diabetes, Pancreas, SIRT1/Akt/PI3K, Vildagliptin, Hormones, Endocrinology, Endocrine system and metabolic diseases

## Abstract

Sirtuin1 (SIRT1) is a histone deacetylase that plays a critical role in insulin sensitivity. Vildagliptin (Vilda) is dipeptidyl peptidase-4 inhibitor approved as oral antidiabetic agent. Docosahexaenoic (DHA) could attenuate hyperglycemia and insulin resistance. The current study was conducted to evaluate the effect of DHA *versus* Vilda on insulin resistance and hyperglycemia in type 2 diabetes (T2D) rat model *via* SIRT1/Akt/PI3K. Eighty male Wistar rats were divided into five groups: normal control, diabetes control (the diabetic rats fed high carbohydrate–high fat diet for four weeks, followed by a single intraperitoneal injection of 35 mg/kg streptozotocin, Vilda + diabetic (diabetic rats received 6 mg/kg vildagliptin), DHA + diabetic (diabetic rats received 300 mg/kg DHA), and DHA only group (normal non-diabetic rats received 300 mg/kg DHA). All treatments were given orally for 4 weeks. Each of Vilda and DHA significantly (p < 0.001) decreased blood glucose (131.40. ± 6.10 mg/L and 137.10 ± 7.37, respectively *vs*. 449.9 ±46.28 1.84 mg/L), increased insulin levels (7.77 ±0.26  µIU/mL and 7.56 ± 0.42, respectively *vs.* 3.86 ± 0.37 ), decreased HOMA-IR (2.52 ± 0.09 and 2.55 ± 0.091, respectively *vs.* 4.26 ± 0.34),   decreased pancreatic malondialdehyde (MDA) (3.84 ± 0.29 nmol/mg protein and 3.18 ± 0.21, respectively *vs.* 6.65 ± 0.71), increased gluthathione (2.18±0.11μmol/mg protein and 2.51±0.09, respectively *vs*. 1.00±0.29), catalase (104.50±6.74 nmol/mg protein and 122.30±6.20, respectively *vs.* 70.30±13.98), increased glutathione peroxidase activity (GPx) (0.76±0.19 U/mg protein and 0.99±0.16, respectively *vs.* 0.47± 0.14) and increased superoxide dismutase activity (SOD) (6.43±1.15 U/mg protein and 8.13±1.16, respectively *vs.* 3.98±1.4) compared with diabetic group. DHA & Vilda significantly (p < 0.001) improved lipid profile (total cholesterol, triglycerides, LDL-C & HDL-C). DHA was superior to Vilda in increasing levels of glutathione (2.51 ± 0.09 µmol/mg protein *vs.* 2.18 ± 0.11), catalase activity (122.30 ± 6.20 nmol/mg protein *vs.* 104.50 ± 6.74 ), SOD activity (8.13±1.16 U/mg *vs.* 6.43±1.15) and GPx activity (0.99±0.16 U/mg protein *vs*. 0.76±0.19). Moreover, both Vilda and DHA significantly increase gene expression of SIRT1, Akt, and PI3K and markedly restored normal pancreatic tissue architecture compared with diabetic control group. DHA was comparable to Vilda as insulinotropic and anti-hyperglycemic agent in T2D rats via activation of SIRT1/Akt/PI3K pathway & reducing oxidative stress.

## Introduction

Type 2 diabetes (T2D) is one of the most common metabolic disorders. Regarding the International Diabetes Federation (IDF), the prevalence of T2D in adults was about 536.6 million people in 2021 (10.5%). Additionally, it is expected that about 783.2 million people will suffer from diabetes worldwide by 2045 (12.2%)^1^**.**

Previous studies revealed that, the phosphatidylinositol 3-kinase/Akt (PI3K/Akt) signaling pathway exerts a critical biological role in the cell such as enhancing viability and attenuating ageing, senescence, and death. It is closely associated with various normal and pathological conditions, and the dysfunction of this pathway is highly correlated with the development of many human diseases, such as leukemia, schizophrenia, and diabetes^2–4^.

Sirtuin1 (SIRT1) is a histone deacetylase which depends on nicotinamide adenine dinucleotide (NAD). SIRT1 plays a critical role in different physiological processes such as metabolism, response to stress, apoptosis, and insulin sensitivity^5,6^. SIRT1 can alleviate insulin resistance through the PI3K/Akt pathway. It deacetylates and activates several key proteins in this pathway, thereby improving insulin sensitivity^5–7^. Recent studies revealed that, activation of SIRT1 is a potential therapeutic target for diabetes management^6,7^.

Dipeptidyl peptidase-4 (DPP4) inhibitors approved their effective role as oral antidiabetic agent. They stimulate insulin secretion while inhibit glucagon release by elevating the endogenous incretin hormones levels^8^. Moreover, DPP4 inhibitors could improve the pancreatic β-cell function via stimulating the proliferation and inhibiting of apoptosis of β-cells^9^. Accordingly, many DPP4 inhibitors are commonly used for the treatment of T2D, including sitagliptin, saxagliptin, and vildagliptin^9^.

Interestingly, it has been found that, vildagliptin improves the sensitivity of β-cell to blood glucose and increases insulin secretion^10,11^. Additionally, it could increase β-cell mass and ratio and augments the pancreatic insulin stores^9^. However, vildagliptin has some uncomfortable adverse effects which include cough, headache, nasopharyngitis, and constipation^11,12^.

Docosahexaenoic acid (DHA) is an essential ω-3 polyunsaturated fatty acid which has beneficial effect in regulating cellular metabolism^13^. It has strong antioxidant activity which can be attributed to the improvement of mitochondrial functions as well as biogenesis. Moreover, DHA has a specific role in relieving symptoms of chronic diseases and improving insulin resistance^14^. In addition, Zhuang et al.^15^ reported that DHA can dramatically attenuate hyperglycemia and insulin resistance in mice with T2D. Interestingly, this beneficial effect of DHA on diabetes is attributed to the alterations of the gut microbiome and the metabolites which links gut to pancreas, adipose, and liver.

While vildagliptin has shown effectiveness in treating T2D, it is associated with several adverse effects such as headache and constipation. As such, alternative therapeutic agents like DHA, have gained attention due to their promising benefits in regulating insulin resistance and improving cellular metabolism^14^.

Insulin resistance in T2D is classically manifested in peripheral tissues such as skeletal muscle, adipose tissue, and liver, rather than in the pancreas. The pancreas, specifically the beta cells, plays a different role: it initially compensates for insulin resistance by increasing insulin production (hyperinsulinemia). Over time, however, beta cells often fail to sustain this high output due to dysfunction and apoptosis, leading to insufficient insulin secretion and the eventual onset of T2D^16,17^. The main objective of the present study was not to directly evaluate peripheral insulin resistance, but rather to investigate the protective effects of DHA and vildagliptin on pancreatic β-cell function and integrity under diabetic conditions. As T2D is strongly associated with β-cell dysfunction driven by glucolipotoxicity, inflammation, oxidative stress, and endoplasmic reticulum stress. Therefore, pancreatic tissue was selected to assess these diabetes-related pathological mechanisms and the potential protective effects of the tested interventions at the level of the β-cell.

Overall, the current study was conducted to investigate the therapeutic efficacy of DHA in comparable to vildagliptin in T2D induced rat model and examine the underlying molecular mechanisms in improving pancreatic function via SIRT1/Akt/PI3K pathway. The study also assessed the baseline metabolic and antioxidant effects of DHA independent of diabetes induction to distinguish between DHA’s general physiological actions and its disease-modifying effects in T2D and to explore its possible preventive potential against insulin resistance and oxidative stress.

## Material and methods

### Animals

Eighty male Wistar rats weighing 105–120 g & five weeks of age were obtained from the National Research Center, Giza, Egypt. The animals were housed under environmental conditions with a temperature of 22 ± 1 °C, 50 ± 10% humidity and 12/12 h light/dark cycle for seven days for acclimatization and allowed free access to water and the normal chow diet (45% carbohydrates, 20% protein, 10% fats & 25% fiber with total energy equal to 3500 kcal/kg).

### Induction of type 2 diabetes and experimental design

After acclimatization, the rats were weighed and divided randomly into two major groups: normal control rats (n = 32) and diabetic rats (n = 48). Animal numbers were chosen with consideration of ethical requirements and the 3Rs principle (replacement, reduction, refinement) to balance statistical sensitivity with minimizing animal use^18^. Normal control rats were maintained on normal chow diet. The diabetic rats fed high carbohydrate–high fat diet (HC/HF) with total energy equal to 4900 kcal/kg containing 60% carbohydrates, 22% saturated fat, 13% protein, 5% fiber for four weeks^19^**,** and followed by a single i.p. injection of 35 mg/kg streptozotocin (Sigma Aldrich, USA) which is freshly prepared in 0.1 M sodium citrate buffer (pH 4.5) to induce T2D in rats^20,21^. After three days of streptozotocin injection, diabetic rats were checked for blood glucose levels from tail tip samples under non-fasted conditions using blood glucosemeter (Accu-check® Roche Diagnostics co., USA). Rats with blood glucose ≥ 300 mg/dL were considered diabetic and were selected to complete the experiment (n = 42)^20^. The final inclusion rate was 42/48 = 87.5% and the exclusion of non-responders were 6 rats. This approach allowed us to preserve statistical power while following ethical principles to minimize unnecessary repeat procedures.

**Fig. 1:**
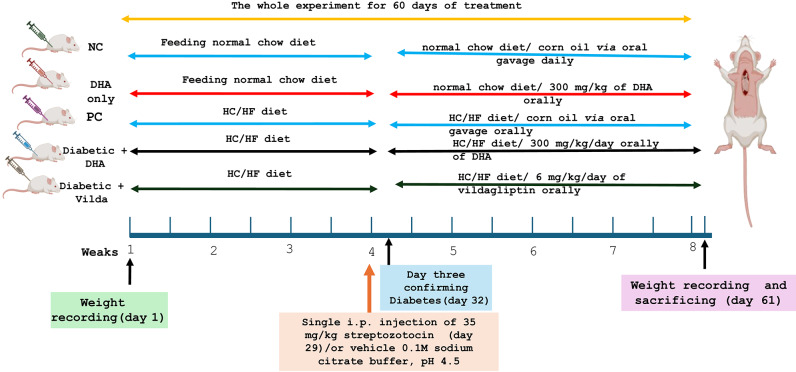
The experimental design of the study. Negative control (NC): non-diabetic rats, positive control (PC): diabetic rats, DHA only: non-diabetic rats treated with 300 mg/kg/day Docosahexaenoic Acid (DHA), diabetic + Vilda.: diabetic rats treated with 300 mg/kg/ day vildagliptin (Vilda), diabetic + DHA: diabetic rats treated with 300 mg/kg/day Docosahexaenoic Acid (DHA). HC/HF : high carbohydrate–high fat diet.

The experimental design was presented in Fig. 1, following the confirmation of diabetes, oral treatments were administered once daily for additional four weeks in the respective groups according to the treatment as follows:

The control group was subdivided into two subgroups (n = 16) to:

**NC group**: Untreated normal control group received the vehicle (a single intraperitoneal injection (i.p.) of 0.1 M sodium citrate buffer, pH 4.5 at the end of week four and corn oil via oral gavage once a day for four weeks.

**DHA only group**: Normal rats received 300 mg/kg of DHA (dissolved in corn oil), orally once a day for four weeks^22^. The volume of corn oil administered was minimal (1 mL/Kg) and it is widely considered metabolically inert at such doses; control animals received equivalent volumes to account for any potential vehicle effects.

Diabetic rats (n-42) were subdivided into three subgroups (n = 14) as follows:

**PC**: Untreated diabetic group received the vehicle (corn oil) orally as a positive control group for additional four weeks parallel with HC/HF diet.

**Diabetic + DHA group**: rats were administered 300 mg/kg/day, orally of DHA (dissolved in corn oil) for additional four weeks parallel with HC/HF diet^22^.

**Diabetic + Vilda**: Rats were received 6 mg/kg/day of vildagliptin, orally (Novartis international, Switzerland) dissolved in phosphate buffered saline (pH = 7.2) for additional four weeks parallel with HC/HF diet^23^.

### Specimen collection

At the end of the experiment, rats were fasted overnight and weighed. Rats then were anaesthetized, and blood was withdrawn via cardiac puncture. Following that, rats were sacrificed, and their pancreas were dissected. Serum samples were collected after centrifugation at 405 × g for determination of fasting glucose, insulin levels, and lipid profile. Pancreas was washed in ice-cold saline and divided into two portions. One portion was kept in formalin solution for further histopathological examination while the other portion was kept at -80 °C for biochemical analysis and gene expression analysis. Pancreatic tissue was homogenized (1: 4 w/v) in 0.1 M phosphate buffer at pH 8.0 using a Polytron homogenizer (Italy). Homogenates were centrifuged at 867 × g for 10 min and supernatants were collected for assessment of oxidants and antioxidant markers.

### Determination of fasting blood glucose, serum insulin level, and HOMA-IR

Fasting blood Glucose level was determined using direct enzymatic colorimetric method (Bio-diagnostic Kit, Cairo, Egypt). Fasting insulin concentration was measured by rat insulin ELISA kit (MLbio Biotechnology, China). Insulin concentration was determined according to manufacturer protocol. Insulin resistance was calculated using homeostasis model assessment-insulin resistance (HOMA-IR) index which was described by Matthews et al*.*^24^ as follows:

$${\mathrm{HOMA}} - {\mathrm{IR}} = \frac{{{\text{FBG }}\left( {{\mathrm{mg}}/{\mathrm{dL}}} \right){\text{ X fasting insulin }}\left( {\mu {\mathrm{IU}}/{\mathrm{mL}}} \right)}}{405}$$

### Determination of fasting serum lipid profile

Serum total cholesterol, HDL-C, LDL-C, VLDL, and triglycerides (TG) were assessed colorimetrically using commercial kits (Biodiagnostic Co., Giza, Egypt)^25^.

### Determination of oxidative stress and antioxidant markers in pancreatic tissue

Pancreatic malondialdehyde (MDA) concentration, marker of lipid peroxidation, was measured colorimetrically using commercial kits (Eagle Biosciences Inc., USA) according to Ohkawa et al*.*^26^. MDA reacted with thiobarbituric acid, and the formed complex was measured at 532 nm. The extent of lipid peroxidation was expressed as MDA (nmoL MDA/mg protein). Further, yellow-coloration of glutathione (GSH) in the pancreatic tissue as a marker of antioxidant capacity, was carried out according to the method of Ellman^27^ using DTNB (2, 2ʹ-dinitro-5,5ʹ-dithiodibenzoic acid) that reacts with SH groups to produce a yellow-colored complex which is measured colorimetrically at 412 nm.

Catalase activity (**CAT, E.C.1.11.1.6**) was assessed in the pancreatic tissue by the method of Aebi^28^ based on tracking the degradation of hydrogen peroxide. One unit of CAT activity was defined as micromoles of H_2_O_2_ decomposed per min.

Glutathione peroxidase (**GPX; EC 1.11.1.9**) activity was determined in the pancreatic tissue homogenate colorimetrically using purchased kits (Biodiagnostic®, Giza, Egypt) by measuring the oxidation of NADPH at 340 nm. One unit of GPX activity is equivalent to the amount of protein that oxidizes 1 mmol/L NADPH per minute^29^. Moreover, superoxide dismutase (**SOD; E.C.1.15.1.1**) activity was determined based on the methods of Pine et al.^30^ by using the commercially available assay kits (Biodiagnostics®, Cairo, Egypt) according to the manufacturer’s protocol. Total proteins were quantified according to Lowry’s method^31^ using bovine serum albumin as standard.

### Quantitative real-time reverse transcriptase polymerase Chain reaction (qRT-PCR)

Gene expressions of SIRT1^32^, Akt, and PI3K were analyzed using RT-PCR and B-actin as housekeeping gene^33^. The total RNA was extracted from pancreatic tissues using RNeasy® Mini kit (Qiagen, Germany), n = 3 (pooled biological replicates, each replicate represents 3 animals)^34^. The concentration of the extracted RNA was measured using Namedrops (Denovix®, U.S.A) and the QuantiTect® reverse transcription kit (Two Step RT-PCR Kit, Qiagen, Germany) was used for cDNA preparation. The produced cDNA was used for PCR with QuantiTect SYBR Green I PCR kit (Qiagen, Germany). Our primers were obtained from Biosearch Technologies, U.S.A and the primer’s sequences are presented in Table 1. The relative gene expression was normalized to β-actin and Livak method (2^-ΔΔCT^) was used for the calculation of fold gene expression^35^.

**Table 1:** Primers’ sequences.

Gene	Sequences
Sirt1	Forward: 5′-GAC GAC GAG GGC GAG GAG -3′) Reverse: 5′- ACA GGA GGT TGT CTC GGT AGC -3′^29^
Akt	Forward: 5′- AAA CCT GGC GGC CAC GCT AC -3` Reverse: 5′- TTG GCC AGG GCC ACC TCC AT -3`
PI3K	Forward: 5′- GGT GCG AGA GGA GTG GAC AA -3` Reverse: 5′- CGG GAC AGG TGG AAG AAC AGC -3`
β-actin	Forward: 5`- GGC TGT ATT CCC CTC CAT CG -3` Reverse: 5`- CCA GTT GGT AAC AAT GCC ATG T -3`^30^

*PI3K* phosphatidylinositol 3-kinase, *SIRT1* sirtuin1, *AKT* protein kinas B.

### Histopathological examination

Tissue specimens of pancreases were fixed in 10% neutral buffered formalin. Transverse pancreas sections were embedded in paraffin. Cross Sects. (4–5 µm thick) of the fixed pancreatic tissues were cut and stained with hematoxylin and eosin (H&E) stain. The images were viewed using Olympus microscope, Japan. Pancreatic histopathological alterations were examined by an expert investigator who was blinded to the experimental design to minimize observer bias.

### Statistical analysis

The sensitivity and post-hoc power calculations were performed using standard two-sample t-test formulas and the G*Power 3.1 software for sample size calculation^36^. Graph-Pad Prism 8.0.1 software (GraphPad, San Diego, CA, USA) was utilized for results analysis. Data are presented as mean ± SD. The statistical comparison between groups was carried out using one-way analysis of variance (ANOVA) followed by Tukey’s multiple comparison test. The significance was set at *p* < 0.05.

## Results

### Effect of DHA and vildagliptin on the weight of diabetic rats

**Fig. 2:**
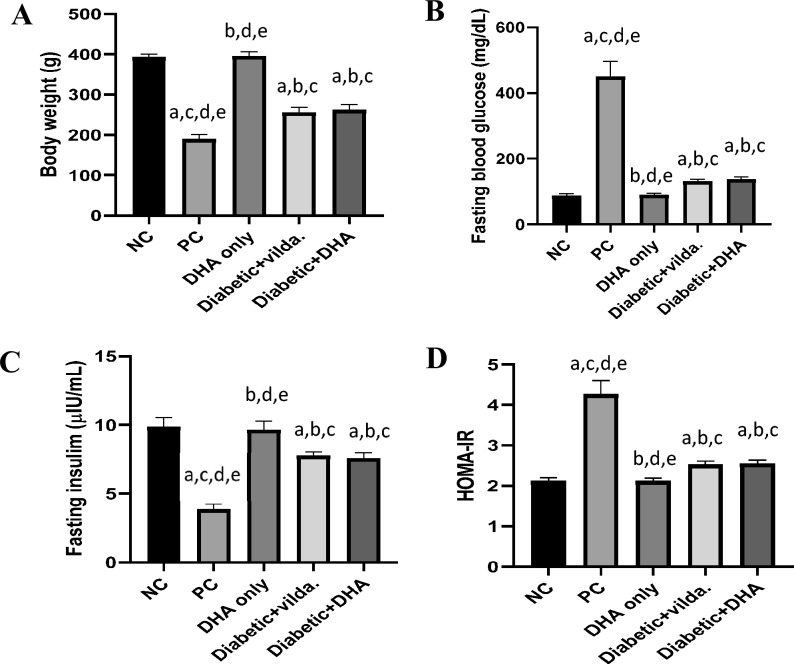
Effect of docosahexaenoic acid (DHA) and vildagliptin on (**A**): Weight of diabetic rats, (**B**): Fasting blood glucose, (**C**): Insulin level, and (**D**): Homeostasis model assessment-insulin resistance (HOMA-IR). Data are presented as mean ± SD, n = 12–16. Negative control (NC): non-diabetic rats, positive control (PC): diabetic rats, DHA only: non-diabetic rats treated with DHA, diabetic + vilda.: diabetic rats treated with vildagliptin, diabetic + DHA: diabetic rats treated with DHA. a: significant versus NC, b: significant versus PC, c: significant *versus* DHA only, d: significant *versus* Diabetic + vilda, and e: significant *versus* Diabetic + DHA. The statistical comparison between groups was carried out using ANOVA followed by Tukey’s multiple comparison test (*p* < 0.05).

Our data showed a significant reduction in body weight of diabetic rats compared to non-diabetic rats in the normal control group (*p* < 0.0001). Meanwhile, diabetic rats treated with DHA or vildagliptin showed significant increase in the body weight compared to untreated diabetic rats (*p* < 0.0001). Interestingly, there was non-significant difference between the weight of normal control group and the non-diabetic rats treated with DHA. Furthermore, the difference between the weights of the diabetic rats treated with DHA and diabetic rats treated with vildagliptin was non-significant (Fig. 2A).

### Effect of DHA and vildagliptin on fasting blood glucose, insulin level, and HOMA-IR

In the current study fasting blood glucose (FBG) and fasting serum insulin were determined at the end of our experiment to investigate the anti-hyperglycemic and insulinotropic effects of DHA. Diabetic rats showed a significant (*p* < 0.0001) increase in the level of FBG (449.90 ± 46.28 mg/dL *vs.* 87.57 ± 5.98 mg/dL) compared to normal control group.

Figure 2B showed that treatment of diabetic rats with vildagliptin or DHA significantly (*p* < 0.0001) decreased the level of FBG (131.40 ± 6.10 mg/L and 137.10 ± 7.37 mg/dL, respectively) compared to the diabetic untreated rats (449.9 ± 46.28 mg/L). Interestingly, there was non-significant difference between the FBG of normal control and nondiabetic rats treated with DHA. Furthermore, non-significant difference was observed between the diabetic rats treated with DHA and vildagliptin (Fig. 2B).

Consistently, fasting serum insulin of the untreated diabetic rats (3.86 ± 0.37 µIU/mL) was significantly lower than that observed in non-diabetic rats in the normal control group (9.86 ± 0.67 µIU/mL, *p* < 0.0001). However, treatment with vildagliptin or DHA significantly (*p* < 0.0001) increased the level of fasting serum insulin (7.77 ± 0.26 µIU/mL and 7.56 ± 0.42 µIU/mL, respectively) compared to diabetic untreated rats. Our results showed non-significant difference of fasting serum insulin between normal control and nondiabetic rats treated with DHA. Moreover, the difference between fasting serum insulin of the diabetic rats treated with DHA and diabetic rats treated with vildagliptin was non-significant (Fig. 2C).

HOMA-IR was calculated for the estimation of insulin resistance in the studied rat groups. Diabetic rats showed significant (*p* < 0.0001) increase in HOMA-IR value (4.26 ± 0.34) compared to the non-diabetic rats (2.12 ± 0.08). Meanwhile, treatment of the diabetic rats with vildagliptin or DHA significantly (*p* < 0.0001) decreased the HOMA-IR value (2.52.10 ± 0.09 and 2.55 ± 0.09, respectively) compared to the diabetic untreated rats.

In addition, there was non-significant difference between the HOMA-IR of normal control group and non-diabetic rats treated with DHA. Further, the difference between HOMA-IR values of the diabetic rats treated with DHA and diabetic rats treated with vildagliptin was non-significant (Fig. 2D).

### Effect of DHA and vildagliptin on the lipid profile of diabetic rats

Table 2 presents the fasting serum lipid profile of studied rats’ group. We can notice that administration of DHA or vildagliptin in diabetic rats significantly decreased total cholesterol (*p* < 0.0001), LDL-c (*p* < 0.0001), VLDL-c (*p* < 0.0001 and *p* < 0.001, respectively), TG (*p* < 0.0001), and the total cholesterol/HDLc risk ratio (*p* < 0.0001) levels in diabetic group compared to untreated diabetic rats. Moreover, the HDL-c levels increased significantly (*p* < 0.0001) in the diabetic rats treated with DHA or vildagliptin compared to untreated diabetic group. More interestingly, DHA was superior to vildagliptin in increasing the level of HDL-c (*p* < 0.01) and in reducing levels of both VLDL-c (*p* = 0.0001) and TG (*p* < 0.0001) in the serum of diabetic rats. Further, administration of DHA to nondiabetic rats improved the lipid profile.

**Table 2:** Lipid profile in the studied rat’s groups.

Criteria	NC	PC	DHA only	Diabetic + Vilda	Diabetic + DHA
Total cholesterol (mg/dL)	145.5 ± 18.0	256.4 ± 30.4^a,c,d,e^	138.0 ± 13.6^b,d,e^	183.7 ± 14.4^a,b,c^	185.8 ± 13.4^a,b,c^
HDL-c (mg/dL)	40.3 ± 2.2	32.2 ± 3.4^a,c,d,e^	44.1 ± 3.3^a,b,d^	39.4 ± 2.5^b,c,e^	43.7 ± 3.1^b,d^
LDL-c (mg/dL)	58.4 ± 33.1	172.9 ± 12.7^a,c,d,e^	83.0 ± 26.5^b,d,e^	114.4 ± 13.9^a,b,c^	116.7 ± 12.6^a,b,c^
VLDL-c (mg/dL)	29.9 ± 5.9	38.6 ± 4.2^a,c,d,e^	13.8 ± 4.6^a,b,d^	28.8 ± 5.2^a,b,c,e^	18.7 ± 5.8^b,d^
TG (mg/dL)	148.4 ± 41.7	265.8 ± 29.4^a,c,d,e^	87.2 ± 33.5^a,b,d^	165.8 ± 33.1^b,c,e^	91.1 ± 46.4^a,b,d^
Risk ratio	3.6 ± 0.5	8.0 ± 0.9^a,c,d,e^	3.1 ± 0.3^b,d,e^	4.7 ± 0.5^a,b,c^	4.3 ± 0.4^b,c^

Data are presented as mean ± SD, n = 12. Negative control (NC): non-diabetic rats, positive control (PC): diabetic rats, DHA only: non-diabetic rats treated DHA, Diabetic + Vilda.: diabetic rats treated with vildagliptin, Diabetic + DHA: diabetic rats treated with the DHA. HDL-c: high density lipoprotein cholesterol; LDL-c: low density lipoprotein cholesterol; TG: triglyceride; VLDL-c: very low-density lipoprotein cholesterol; Risk ratio: total cholesterol/HDLc. a: signifiant *versus* NC, b: signifiant *versus* PC, c: signifiant *versus* DHA only, d: signifiant versus Diabetic + Vilda, and e: signifiant *versus* Diabetic + DHA. The statistical comparison between groups was carried out using ANOVA followed by Tukey’s multiple comparison test (*p* < 0.05).

### Effect of DHA and vildagliptin on oxidative stress in the pancreatic tissue of studied groups

**Fig. 3:**
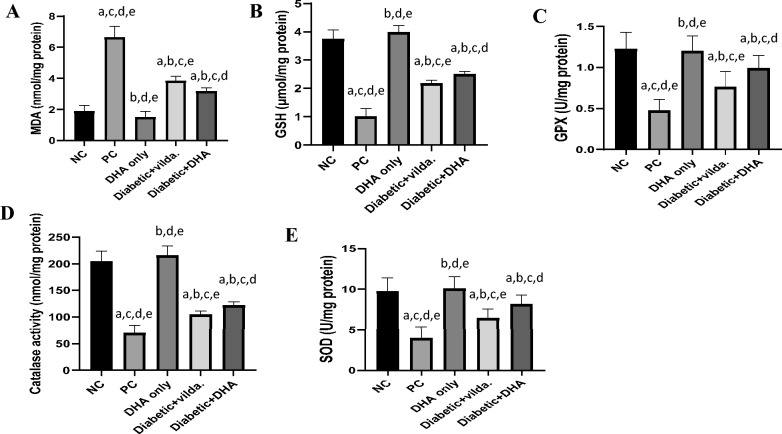
Effect of docosahexaenoic acid (DHA) and vildagliptin on (**A**): Malondialdehyde (MDA) level, (**B**): Glutathione (GSH) level, (**C**): Glutathione peroxidase (GPX) activity, (**D**): Catalase activity, and (**E**): Superoxide dismutase (SOD) activity in the pancreatic tissue of the studied rat’s groups. Data are presented as mean ± SD, n = 12–16. Negative control (NC): non-diabetic rats, positive control (PC): diabetic rats, DHA only: non-diabetic rats treated with DHA, diabetic + vilda.: diabetic rats treated with vildagliptin, diabetic + DHA: diabetic rats treated with DHA. a: significant versus NC, b: significant versus PC, c: significant versus DHA only, d: significant versus Diabetic + vilda, and e: significant versus Diabetic + DHA. The statistical comparison between groups was carried out using ANOVA followed by Tukey’s multiple comparison test (*p* < 0.05).

Our current study revealed that induction of T2D significantly increased the lipid peroxidation marker (MDA) in the pancreatic tissue. The concentration of MDA was significantly higher (*p* < 0.0001) in the pancreas of diabetic rats (6.65 ± 0.71 nmol/mg protein) compared to its concentration in the pancreas of normal control group (1.89 ± 0.38 nmol/mg protein) (Fig. 3A). Meanwhile, treatment of the diabetic rats with vildagliptin or DHA significantly (*p* < 0.0001) decreased MDA levels (3.84 ± 0.29 nmol/mg protein and 3.18 ± 0.21 nmol/mg protein, respectively) compared to the diabetic untreated rats. Moreover, it can be noticed that the concentration of MDA in the pancreases of the diabetic rats treated with DHA was significantly lower than its concentration in the pancreas of diabetic rats treated with Vilda (*P* < 0.005) (Fig. 3A).

On the other hand, the concentration of GSH and catalase activity were significantly lowered (*p* < 0.0001) in the pancreas of diabetic rats (1.00 ± 0.29 µmol/mg protein and 70.30 ± 13.98 nmol/mg protein**,** respectively) compared to normal control group (3.75 ± 0.32 µmol/mg protein and 204.10 ± 15.42 nmol/mg protein**,** respectively). Meanwhile, treatment of the diabetic rats with Vilda or DHA significantly (*p* < 0.0001) increased the level of GSH (2.18 ± 0.11 µmol/mg protein and 2.51 ± 0.09 µmol/mg protein, respectively) as well as catalase activity (104.50 ± 6.74 nmol/mg protein and 122.30 ± 6.20 nmol/mg protein, respectively) compared to the diabetic untreated rats (Fig. 3B & D).

Further, the enzyme activities of GPX and SOD were significantly lowered (*p* < 0.0001) in the pancreas of diabetic rats (0.47 ± 0.14 U/mg protein and 3.98 ± 1.40 U/mg protein**,** respectively) compared to normal control group (1.23 ± 0.20 U/mg protein and 9.73 ± 1.7 U/mg protein**,** respectively). However, treatment of the diabetic rats with Vilda or DHA significantly increased the enzyme activity of GPX (0.76 ± 0.19 U/mg protein; *p* < 0.01 and 0.99 ± 0.16 U/mg protein;* p* < 0.0001, respectively) and SOD (6.43 ± 1.15 U/mg protein;* p* < 0.001 and 8.13 ± 1.16 U/mg protein;* p* < 0.0001, respectively) compared to the diabetic untreated rats (Fig. 3C& D).

In addition, we noticed that the level of GSH and the activity of GPX, catalase, and SOD in the pancreas of diabetic rats treated with DHA were significantly higher than their levels in the diabetic rats treated with vildagliptin (*p* < 0.05) (Fig. 3B-E). Furthermore, there was no significant difference in the levels of pancreatic MDA, GSH, and catalase activity between nondiabetic rats in the negative control group and nondiabetic rats treated with DHA (Fig. 3).

### Effect of DHA and vildagliptin on gene expression of SIRT1, PI3K, and Akt

**Fig. 4:**
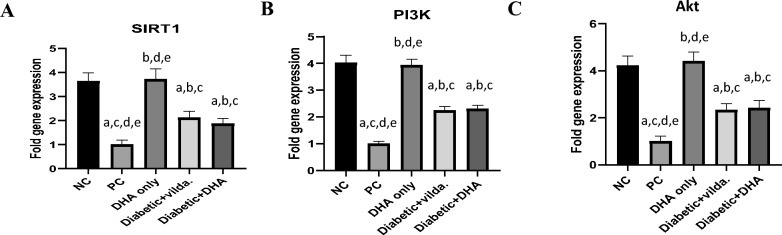
Fold gene expression analysis in the pancreas of the studied rat’s groups (**A**): Sirtuin1 (SIRT1), (**B**): Phosphatidylinositol 3-kinase (PI3K), and (**C**): Akt. Data are presented as mean ± SD, n = 3 (pooled biological replicates, each replicate represents 3 animals). Negative control (NC): non-diabetic rats, positive control (PC): diabetic rats, DHA only: non-diabetic rats treated with DHA, diabetic + vilda.: diabetic rats treated with vildagliptin, diabetic + DHA: diabetic rats treated with DHA. a: significant versus NC, b: significant versus PC, c: significant versus DHA only, d: significant versus Diabetic + vilda, and e: significant versus Diabetic + DHA. The statistical comparison between groups was carried out using ANOVA followed by Tukey’s multiple comparison test (*p* < 0.05).

Induction of T2D caused significant reduction (*p* < 0.0001) in the gene expression of SIRT1, PI3K, and Akt in the pancreatic tissue of diabetic rats (1.00 ± 0.19 fold, 1.00 ± 0.16 fold, and 1 ± 0.23 fold**,** respectively) compared to their gene expression in the pancreas of nondiabetic rats in the normal control group (3.63 ± 0.36 fold, 4.03 ± 0.48 fold, and 4.22 ± 0.42 fold**,** respectively) (Fig. 4). Meanwhile, the treatment of the diabetic rats with Vilda or DHA significantly increased the gene expression of SIRT1 (2.11 ± 0.28 fold, *p* < 0.05 and 1.86 ± 0.23 fold, *p* < 0.05, respectively), PI3K (2.25 ± 0.26 fold, *p* = 0.0054 and 2.30 ± 0.24 fold, *p* < 0.005, respectively), and Akt (2.33 ± 0.28 fold, *p* < 0.005 and 2.41 ± 0.33 fold, *p* < 0.005 respectively) compared to the diabetic untreated rats (Fig. 4).

Furthermore, our data showed non-significant differences in gene expression of pancreatic SIRT1, Akt, and PI3K between the diabetic rats treated with DHA and vildagliptin. Moreover, the difference in the pancreatic gene expression of SIRT1, Akt, and PI3K between normal control group and nondiabetic rats treated with DHA was non-significant (Fig. 4).

### Effect of DHA and vildagliptin on the pancreas histopathology

**Fig. 5:**
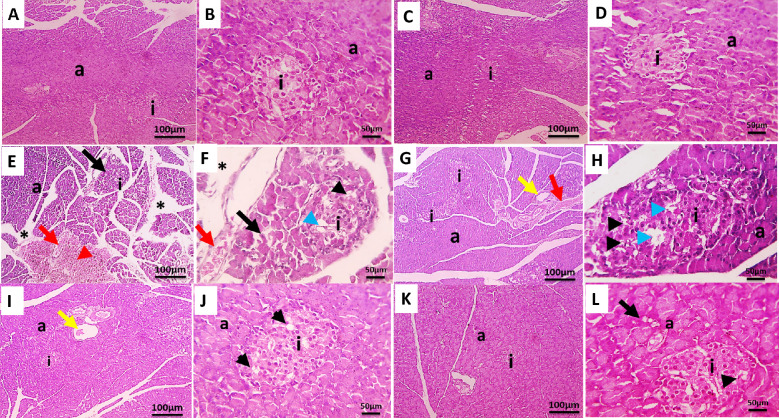
Microscopic pictures of HE stained pancreatic sections of the studied rat’s groups. Pancreatic sections from normal control group (**A** and **B**) and control normal group treated with docosahexaenoic acid (**C** and **D**) showing non histopathological alteration with normal histological structure of the acini (a) as the exocrine portion and the islands of Langerhans (i) containing α-cells and β-cells as the endocrine portion. (**E**—**H**): Pancreatic sections from control diabetic group showing interstitial edema (*), shrinkage of the acini (a) (black arrow), loss of cells in islands of Langerhans (i) (blue arrowheads), vacuolated β-cells (black arrowheads), congestion (red arrow), and hemorrhage (red arrowhead). (**I** and **J**): Pancreatic sections from diabetic rats treated with docosahexaenoic acid showing better histology of acini (a), dilated pancreatic duct (yellow arrow), and vacuolated β-cells (black arrowheads) in islands of Langerhans (i). (**K** and **L**): Pancreatic sections from diabetic rats treated with vildagliptin showing better histology of acini (a), mildly vacuolated β-cells (black arrowheads) in islands of Langerhans (i), and very mildly vacuolated epithelial cells lining (black arrow) few acini (a). Low magnification X: 100, bar 100 µm and high magnification X: 400, bar 50 µm.

The most extensive pathological changes were detected in the pancreatic tissues based on the histopathological examination of four pancreatic sections from each studied group. Table 3 presents the semiquantitative pancreatic histopathological alterations in the studied group. The microscopic examination of H&E-stained pancreatic sections from normal control group and normal group treated with DHA revealed non histopathological alteration and normal histological structure of the acini as the exocrine portion and the islands of Langerhans containing α-cells and β-cells as the endocrine portion (Fig. 5A-D). On the other hand, the pancreatic sections from diabetic group showed interstitial edema, shrinkage of the acini, loss of cells in islands of Langerhans, vacuolated β-cells, congestion, and hemorrhage (Fig. 5E-H). Meanwhile, pancreatic sections from diabetic rats treated with DHA showed better histology of acini, dilated pancreatic duct, and vacuolated β-cells in islands of Langerhans (Fig. 5 I and J). Consistently, the pancreatic sections from diabetic rats treated with vildagliptin revealed better histology of acini, mildly vacuolated β-cells in islands of Langerhans, very mildly vacuolated epithelial cells lining few acini (Fig. 5K and L).

**Table 3:** Semiquantitative pancreatic histopathological alterations in studied groups.

Criteria 0–3	NC	PC	DHA only	Diabetic + Vilda	Diabetic + DHA
Interstitial edema	0.0 ± 0.0^b^	1.0 ± 0.4^a^	0.0 ± 0.0^b^	0.0 ± 0.0 ^b^	0.0 ± 0.0^b^
Shrinkage of the acini	0.0 ± 0.0^b^	0.8 ± 0.4^a^	0.0 ± 0.0^b^	0.0 ± 0.0^b^	0.0 ± 0.0^b^
Vacuolation in B cells	0.0 ± 0.0^b^	2.5 ± 0.2^a^	0.0 ± 0.0^b^	1.0 ± 0.0^ab^	1.5 ± 0.2^a^
Dilation of pancreatic ducts	0.0 ± 0.0^b^	1.6 ± 0.2^a^	0.0 ± 0.0^b^	0.0 ± 0.0^b^	0.8 ± 0.2^ab^
Congestion	0.0 ± 0.0^b^	1.6 ± 0.2^a^	0.0 ± 0.0^b^	0.0 ± 0.0^b^	0.0 ± 0.0^b^
Hemorrhage	0.0 ± 0.0^b^	1.8 ± 0.3^a^	0.0 ± 0.0^b^	0.0 ± 0.0^b^	0.0 ± 0.0^b^

Data are presented as mean ± SD, n = 12. Negative control (NC): non-diabetic rats, positive control (PC): diabetic rats, DHA only: non-diabetic rats treated DHA, diabetic + vilda.: diabetic rats treated with the vildagliptin, diabetic + DHA: diabetic rats treated with the DHA. a significant versus NC; b significant versus PC.

## Discussion

Type 2 diabetes (T2D) is one of the most common metabolic disorders. The prevalence of T2D is increasing all over the world^1^**.** Furthermore, uncontrolled T2D contributes to various micro- and macro-vascular complications as well as to early mortality (Rahman et al., 2015).

Vildagliptin is a commonly used as oral antidiabetic agent that elevates incretin hormones via inhibition of DPP-4 enzyme^8^. Thus, vildagliptin improves both fasting blood glucose (FBG) as well as postprandial blood glucose (PBG)^37^. Interestingly, it has been found that, vildagliptin improves the sensitivity of β-cell to blood glucose and increases insulin secretion^10,11^. Additionally, it could increase β-cell mass and ratio and augments the pancreatic insulin stores^8^. However, as a synthetic pharmacological agent, vildagliptin has some side effects including headache, fever, and constipation^11,12^**.** Previous studies displayed the ability of many natural products, including omega-3 & DHA to reduce blood glucose levels in diabetes^15,38,39^. This study was conducted to compare the therapeutic effects of DHA and vildagliptin (DPP4 inhibitor) on T2D rat model and to clarify the molecular mechanisms underlying DHA action.

Experimental T2D was induced in the present study in male Wistar rats by high carbohydrate–high fat diet (HC/HF) followed by single i.p. of streptozotocin. This model is now widely referred to as a T2D model, it mimics the natural progression of type 2 diabetes, transitioning from insulin resistance/prediabetes to overt diabetes with hypoinsulinemia within a shortened timeframe^40^. In the present study, rats were fed a high-fat diet for four weeks to induce insulin resistance which mimics the early stage. Subsequently, animals received a single dose of streptozotocin (35 mg/kg), leading to sustained hyperglycemia within three days post-injection that mimics late stage. This impaired glucose clearance & hypoinsulinemia were due to β-cell damage as evidenced diabetic rats.

In the current study, the diabetes control group showed a significant elevation of body weight by HC/HF which indicates obesity. Also, elevated levels FBG and HOMA-IR as well as reduction in fasting serum insulin level compared to normal control. Diabetes induction was also evidenced by the histopathological changes of the pancreas which showed interstitial edema, shrinkage of the acini, loss of cells in islands of Langerhans, vacuolated β-cells, congestion, and hemorrhage. Streptozotocin (STZ) selectively targets and destroys pancreatic beta cells by entering through GLUT2 transporters, causing DNA damage, oxidative stress, and ultimately cell death (necrosis/apoptosis), leading to severe insulin deficiency and diabetes. This toxicity disrupts insulin secretion, reduces insulin content, and impairs overall islet function^41^

Rats fed HFD develop hyperlipidemia before STZ injection. The high-fat feeding elevates circulating free fatty acids (FFAs). FFAs accumulate in β-cells, impairing insulin secretion and promoting apoptosis. In addition: lipid metabolites such as ceramides & diacylglycerol can interfere with insulin signaling, worsening insulin resistance^42^. In our rat model of T2D, lipotoxicity contributes to β-cell dysfunction after STZ exposure, accelerating the diabetic phenotype. Dyslipidemia exacerbates insulin resistance and promotes lipotoxicity in peripheral tissues^40^. The combined effect of dyslipidemia and STZ-induced β-cell damage leads to stable hyperglycemia, therefore this model mirrors human T2DM, where abnormal lipid metabolism is a key driver of complications.

Our findings demonstrated that treatment of diabetic rats with vildagliptin or DHA significantly improved the lipid profile and decreased FBG as well as HOMA-IR while fasting serum insulin and body weight were increased compared to untreated diabetic rats in the positive control group. Interestingly, we found for the first time that treatment of diabetic rats with DHA decreased the level of FBG and increased fasting serum insulin to a level comparable to that of vildagliptin treatment. Further, DHA was superior to vildagliptin in increasing the level of HDL-c and reducing the levels of both VLDL-c and TG in the serum of diabetic rats.

We observed that administration of DHA to non-diabetic rats did not affect fasting blood glucose, fasting serum insulin, or HOMA-IR. These findings highlight the normoglycemic nature of DHA, indicating its ability to counteract hyperglycemia under diabetic conditions without inducing hypoglycemia in healthy animals. However, administration of DHA to nondiabetic rats improved the fasting serum lipid profile.

Our results were in accordance with Zhuang et al*.*^15^ which revealed that supplementation of DHA attenuates insulin resistance and hyperglycemia in mice with T2D. Furthermore, Werida et al.^38^, reported that administration of omega-3 fatty acids for three months to diabetic patients significantly decreases FBG and HOMA-IR as well as improves lipid profile.

Persistent hyperglycemia in diabetes induces oxidative stress via mitochondrial dysfunction and subsequent enhanced reactive oxygen species (ROS) production. Excessive ROS may lead to severe oxidative damage^42^. DHA has promising antioxidant activities which have been extensively studied. DHA significantly decreases both cellular and mitochondrial ROS. It also significantly increases the total antioxidant capacity. Furthermore, it increases the gene expression and activities of the major antioxidant enzymes^13^.

In the current study, induction of T2D significantly increased the lipid peroxidation marker (MDA) and decreased antioxidant markers (catalase activity and GSH level) in the pancreatic tissues of rats. Treatment with either vildagliptin or DHA significantly reversed the effects of diabetes induction on the antioxidant and oxidative stress markers in the pancreatic tissue. In addition, our study highlighted that the level of MDA in the pancreas of diabetic rats treated with DHA were significantly lower than their level in the diabetic rats treated with vildagliptin (*p* < 0.005). Moreover, the enzyme activity of catalase, GPX, and SOD as well as the level of GSH in the pancreas of diabetic rats treated with DHA were significantly higher than their levels in the diabetic rats treated with vildagliptin. This indicated that DHA was superior to vildagliptin in reducing oxidative stress in the pancreatic tissue of diabetic rats.

We can also notice that administration of DHA to nondiabetic rats did not affect the levels MDA and GSH. Further, the enzyme activity of GPX, SOD, and catalase in nondiabetic rats treated with DHA showed non-significant difference from the negative control group. These findings shine a light on the ability of DHA to counteract the increase in the oxidative stress caused by hyperglycemia in diabetes without affecting nondiabetic normoglycemic rats.

Our results were supported by the study accomplished by Aghahoseini et al*.*^23^ who reported that vildagliptin significantly reduced the level of MDA and increased catalase and glutathione oxidase levels in the renal tissue of T2D rats. Furthermore, An et al.^14^ revealed the potent antioxidant capacity of DHA in diabetic rats which demonstrated by elevating catalase, glutathione peroxidase, and superoxide dismutase activities as well as reducing MDA level in the liver tissue.

The HF/HC diet–low-dose STZ model induces insulin resistance followed by partial β-cell dysfunction, mimicking type 2 diabetes. Oxidative stress in this model reflects the combined effects of glucotoxicity, lipotoxicity, and STZ-induced β-cell injury. In the present study, pancreatic oxidative stress markers (MDA, CAT, SOD, GPx, and GSH) were measured, and both DHA and Vildagliptin significantly reduced MDA and enhanced antioxidant defenses, confirming their protective effects on β-cells and supporting their translational relevance.

Phosphatidylinositol 3-kinase (PI3K) is an enzyme which is activated by the insulin receptor signaling and could regulate many cellular processes, including growth, metabolism, insulin sensitivity, and glucose homeostasis^2,3^. Insulin triggers the phosphorylation of its receptor, which in turn leads to the phosphorylation of insulin receptor substrates at tyrosine residues. This activation process stimulates phosphatidylinositol-3 kinase (PI3K) and the subsequent conversion of phosphatidylinositol-4,5-bisphosphate (PIP2) into phosphatidylinositol-3,4,5-trisphosphate (PIP3). Protein kinase B (Akt) is then recruited to the membrane, where it activates the mTOR protein complex. This cascade of events results in a variety of metabolic effects, including the regulation of nutrient uptake and maintenance of glucose homeostasis^2,3^.

Multiple studies have demonstrated that PI3K/Akt pathway plays a vital role in the maintenance of glucose homeostasis^4^**.** Accordingly, PI3K/Akt pathway could be considered as a new target for the development of recent hypoglycemic agents.

Sirtuin1 (SIRT1) is a histone deacetylase which depends on NAD; SIRT1 interplay significantly in different physiological processes such as regulation of glucose metabolism and insulin sensitivity^5,6^. SIRT1 can alleviate insulin resistance through the PI3K/Akt pathway. It deacetylates and activates several key proteins in this pathway, thereby improving insulin sensitivity^6,7^. Recent studies revealed that, activation of SIRT1 is a potential therapeutic target for diabetes management^6,7^.

Regarding our RT-PCR findings, the induction of T2D caused significant reduction (*p* < 0.0001) in the gene expression of SIRT1, PI3K, and Akt in the pancreatic tissue of diabetic rats in the positive control group compared to their gene expression in the pancreas of nondiabetic rats in the normal control group. Meanwhile, treatment of diabetic rats with vildagliptin or DHA significantly increased the gene expression of SIRT1 (*p* < 0.05), PI3K (*p* < 0.01), and Akt (*p* < 0.005) compared to the diabetic untreated rats in the positive control group.

Furthermore, our data showed non-significant differences in the gene expression of pancreatic SIRT1, Akt, and PI3K between diabetic rats treated with DHA and diabetic rats treated with vildagliptin. Moreover, the difference in pancreatic gene expression of SIRT1, Akt, and PI3K between non-diabetic rats in the control group and non-diabetic rats treated with DHA was not statistically significant. These findings indicate that DHA does not overstimulate the normally regulated SIRT1/Akt/PI3K pathway in healthy pancreas but is able to reactivate this pathway when suppressed under diabetic conditions. The inclusion of the DHA-only group therefore confirmed that the observed therapeutic benefits of DHA are disease-specific rather than a consequence of nonspecific pathway activation, while also highlighting its potential preventive role through maintaining redox and metabolic balance.

Our results showed that gene expressions of SIRT1, PI3K & Akt were increased after treatment with VILDA & DHA. These results were in accordance with Sayed et al*.*^43^, who revealed that VILDA could attenuate the induced-Huntington’s disease in rat model via activating PI3K/Akt pathway. Moreover, the study conducted by Du et al*.*^44^ revealed that DHA- enriched phospholipids alleviate intestinal barrier injury by activating the Nrf2 antioxidant pathway via up-regulating SIRT1.

In the current study, RT-PCR was used to assess transcriptional changes in SIRT1/PI3K/Akt key signaling molecules, providing preliminary evidence of pathway modulation by DHA and Vildagliptin. RT-PCR findings provide the involvement of the SIRT1/PI3K/Akt pathway in pancreatic tissue under diabetic conditions and reveal that the beneficial therapeutic effect of DHA on diabetes could be mediated by its ability to activate SIRT1/Akt/PI3K pathway.

The biochemical results were largely consistent with our observed histopathological findings as the sections of the pancreas from the diabetic rats treated with vildagliptin or DHA showed marked improvement in the diabetes induced-histopathological changes and restored the normal histological architecture of islets of Langerhans.

## Conclusion

DHA has a promising insulinotropic and anti-hyperglycemic effect in T2D rats. Our results showed that DHA was comparable to vildagliptin in lowering blood glucose level and HOMA-IR value and increasing insulin secretion. Moreover, DHA was superior to vildagliptin in reducing oxidative stress and improving lipid profile. The beneficial therapeutic effect of DHA on diabetes could be attributed to its strong antioxidant activity and its ability to activate SIRT1/Akt/PI3K pathway and restore the normal histological architecture of the pancreas. DHA could be used as adjuvant therapy with other synthetic drugs for the treatment of T2D patients. However, further clinical studies are recommended to investigate the beneficial therapeutic efficacy of DHA over vildagliptin.

### Limitation of the study

The observed benefits of DHA are primarily related to β-cell protection against oxidative stress and pancreatic insulin signaling. These findings do not claim full equivalence to human T2DM and further studies investigating skeletal muscle, liver, and adipose tissue insulin signaling are required to strengthen translational relevance. Also, the animal model used for induction of T2D and insulin resistance limits direct clinical extrapolation. Finally, a limitation of the present study is that Akt and PI3K signaling was assessed only at the gene expression level using RT-PCR. Since these proteins are mainly regulated post-translationally (e.g., via phosphorylation), mRNA levels may not fully reflect pathway activation. Future studies examining protein expression and activation, such as Western blot analysis of phosphorylated Akt and PI3K, are warranted to confirm the mechanistic effects of DHA and Vildagliptin on the SIRT1/Akt/PI3K pathway in insulin-sensitive tissues such as skeletal muscle, liver, and adipose tissue to strengthen mechanistic and translational relevance.

## Data Availability

Data were depositing in a public repository and any one can access it using this link ([Data Analysis of vildaglibtin + docosa](https:/pharmtantaedu-my.sharepoint.com/:f:/g/personal/naglaa_khedr_pharm_tanta_edu_eg/EqONO5Ti0zBEr5rSQAbKuNQBjXotoZv81TH2z_yEmBlkig?e = ADPqUi)) or from the corresponding author: Naglaa Khedr. Email: [naglaa.khedr@pharm.tanta.edu.eg.](mailto:naglaa.khedr@pharm.tanta.edu.eg) Al-Baher Street, Medical compound, Faculty of Pharmacy, Tanta University, P.O; 31,527, Egypt.

## Abbreviations

DHA: Docosahexaenoic acid
DPP4: Dipeptidyl peptidase-4
FBG: Fasting blood glucose
GSH: Glutathione
H&E: Hematoxylin and eosin
HOMA-IR: Homeostasis model assessment-insulin resistance
IDF: International diabetes federation
MDA: Malondialdehyde
PGG: Postprandial blood glucose
PI3K: Phosphatidylinositol 3-kinase
ROS: Reactive oxygen species
T2D: Type 2 diabetes
TG: Triglycerides
GPx: Glutathione peroxidase
SOD: Super oxide dismutase
